# Blocking Formation of the Stable HIV Reservoir: A New Perspective for HIV-1 Cure

**DOI:** 10.3389/fimmu.2019.01966

**Published:** 2019-08-22

**Authors:** Nilu Goonetilleke, Genevieve Clutton, Ron Swanstrom, Sarah B. Joseph

**Affiliations:** ^1^Department of Microbiology & Immunology, University of North Carolina at Chapel Hill, Chapel Hill, NC, United States; ^2^UNC HIV-1 Cure Center, University of North Carolina at Chapel Hill, Chapel Hill, NC, United States; ^3^Department of Biochemistry and Biophysics, University of North Carolina at Chapel Hill, Chapel Hill, NC, United States; ^4^Lineberger Cancer Center, University of North Carolina at Chapel Hill, Chapel Hill, NC, United States

**Keywords:** CD4^+^ T cell, HIV-1, memory, latency, reservoir, IL-7, CD127

## Abstract

Recent studies demonstrate that the stable HIV-1 reservoir in resting CD4^+^ T cells is mostly formed from viruses circulating when combination antiretroviral therapy (ART) is initiated. Here we explore the immunological basis for these observations. Untreated HIV-1 infection is characterized by a progressive depletion of memory CD4^+^ T cells which mostly express CD127, the α chain of the IL-7 receptor (IL-7R). Depletion results from both direct infection and bystander loss of memory CD4^+^ T cells in part attributed to dysregulated IL-7/IL-7R signaling. While IL-7/IL7R signaling is not essential for the generation of effector CD4^+^ T cells from naïve cells, it is essential for the further transition of effectors to memory CD4^+^ T cells and their subsequent homeostatic maintenance. HIV-1 infection therefore limits the transition of CD4^+^ T cells from an effector to long-lived memory state. With the onset of ART, virus load (VL) levels rapidly decrease and the frequency of CD127^+^ CD4^+^ memory T cells increases, indicating restoration of effector to memory transition in CD4^+^ T cells. Collectively these data suggest that following ART initiation, HIV-1 infected effector CD4^+^ T cells transition to long-lived, CD127^+^ CD4^+^ T cells forming the majority of the stable HIV-1 reservoir. We propose that combining ART initiation with inhibition of IL-7/IL-7R signaling to block CD4^+^ T cell memory formation will limit the generation of long-lived HIV-infected CD4^+^ T cells and reduce the overall size of the stable HIV-1 reservoir.

## Key Points

- Both the long-lived defective and replication competent HIV-1 reservoirs in CD4^+^ T cells form near the time of ART initiation.
- The replication competent HIV-1 reservoir in CD4^+^ T cells is stable under ART.
- Memory CD4^+^ T cells which mostly express the IL-7 receptor (IL-7R) α chain, CD127 are profoundly depleted during untreated HIV-1 infection.
- HIV-1 infection disrupts IL-7/IL-7R signaling and CD4^+^ T cell memory formation.
- CD4^+^ T cell memory formation and IL-7/IL-7R signaling is restored following ART initiation.
- Blocking IL-7/IL-7R signaling limits CD4^+^ T cell memory generation.
- Blocking IL-7/IL-7R signaling at ART initiation may limit the transition of HIV-infected cells to long-lived memory CD4^+^ T cells, decreasing the overall size of the stable HIV-1 reservoir.

## The HIV-1 Reservoir Is Established Around the Time of ART Initiation

Untreated HIV-1 infection is characterized by continual viral replication and evolution. However, two papers by independent groups, combining HIV-1 sequencing approaches and longitudinal sampling of persons living with HIV (PLWH), before and after ART initiation, concluded that the majority of the HIV-1 reservoir is formed around the time of ART initiation (1, 2). Brodin et al. used an Illumina™ deep sequencing approach to compare p17*gag* sequences in plasma virus RNA (vRNA) collected longitudinally for at least the first 5 years after diagnosis but before ART (pre-ART) to proviral DNA isolated from peripheral blood mononuculear cells (PBMCs) after at least 2 years of suppressive ART. In this study of 10, mostly HIV-1 clade B-infected Swedish individuals (9 male, 1 female), phlyogenetic analysis found that ~60% of the post-ART DNA sequences were most similar to RNA variants that were present in the plasma just prior to ART initiation (1).

The HIV-1 DNA reservoir is dominated by defective proviruses (3–5), therefore Brodin et al.'s study did not provide information on the timing of establishment of the stable *replication competent* reservoir, which is a primary source of rebounding virus following ART interruption. This question was addressed by Abrahams, Joseph *et a*l., who used samples from nine HIV-1 clade C-infected South African women to compare pre-ART HIV-1 RNA (longitudinally sampled from the plasma during 2.7–6.9 years of untreated infection) to replication competent HIV-1 in resting CD4^+^ T cells obtained post-ART (after 4.7–6.1 years of ART) (2). Briefly, MiSeq with PrimerID (6, 7) was used to sequence five regions of the HIV-1 genome (*gag, nef*, and three regions in *env*) in pre-ART vRNA. In addition, resting CD4^+^ T cells (CD25-CD69-HLADR-) isolated post-ART were cultured after stimulation (PHA, IL-2, and allogenic PBMC) and the PacBio platform was used to generate 5′ and 3′ half genome sequences from the HIV-1 RNA produced by the resting CD4^+^ T cells. Phylogenetic analyses of these sequences revealed that a median of 78% of outgrowth viruses were most genetically similar to viruses circulating in the year before ART. Together these studies show that both the defective (3) and replication competent HIV-1 reservoirs (2) form near the time of ART initiation.

These independent observations, made in distinct clinical cohorts, are most simply explained by ART indirectly increasing the half-life of cells harboring integrated virus resulting in a stable reservoir. Given that most studies agree that virus evolution does not occur on ART (1, 8–10), the work of Abrahams, Joseph and colleagues identifies a narrow time window immediately after therapy initiation in which the majority of the stable/long-lived HIV-1 reservoir is established. This suggests that strategies to *limit the formation* of the stable HIV-1 reservoir could be combined with ART initiation, when patients are receiving intense clinical care. Preventing generation of long-lived latently infected CD4^+^ T cells should result in a smaller HIV-1 reservoir, providing a less intractable target for curative approaches. Reducing the size of the HIV-1 reservoir may also reduce ongoing immune senescence and HIV-1 co-morbidities experienced by PLWH on ART.

**Figure 1:**
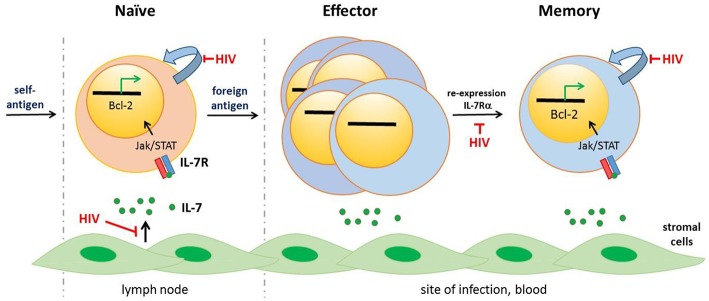
CD4^+^ T cell lineage differentiation is impaired following HIV-1 infection. IL-7, that is mostly produced by stromal cells, binds the IL-7 receptor (IL-7R) comprising CD132 and IL-7Rα (CD127) initiating signaling pathways including as Jak/STAT5 signaling and expression of anti-apoptopic genes, including Bcl-2. Naïve CD4^+^ T cells, selected against self-antigens, express the IL-7R. IL-7/IL-7R signaling is necessary for homeostatic turnover of naïve T cells (curved blue arrow). Following priming by foreign antigens, CD4^+^ T cells undergo significant transcriptional and phenotypic changes, including downmodulation of the IL-7R. These effector CD4^+^ T cells express activation markers (not shown), undergo rapid division and exit the lymph node to home to the site of infection. Most effector CD4^+^ T cells undergo apoptopic death; a subset however re-express the IL-7R and induce expression of anti-apoptopic genes. These memory cells are long-lived, undergoing slow mitotic division/homeostatic proliferation. HIV-1 infection drives ongoing expansion of effector CD4^+^ T cells. In addition, dysregulated IL-7/IL-7R signaling resulting from HIV-induced immune activation impairs several stages of CD4^+^ T cell lineage—naïve CD4^+^ T cell survival, generation of long-lived memory CD4^+^ T cells and homeostatic proliferation of naïve and memory CD4^+^ T cells. Other cytokines, notably IL-2 and IL-15, contribute to CD4^+^ T cell differentiation and homeostasis [not illustrated, reviewed in (11)].

Here, we propose that establishment of the HIV-1 reservoir at the time of ART initiation is driven by the restoration of IL-7/IL-7R signaling that increases CD4^+^ T cell transition to long-lived memory cells (Figure 1). In this review, we discuss how untreated HIV-1 infection disrupts CD4^+^ T cell homeostasis and how homeostasis is subsequently restored on ART, consistent with ART facilitating the establishment of the majority of the stable HIV-1 reservoir in long-lived CD4^+^ T cells. We propose that a novel approach to complement existing HIV-1 therapies is to minimize establishment of the HIV-1 reservoir at ART initiation by blocking the IL-7/IL-7R-mediated CD4^+^ T cell memory transition until viremia is cleared and the immune environment transitions to a less inflammatory state.

## ART-Suppressed Individuals Harbor a Stable HIV-1 Reservoir

ART is highly effective at stopping new rounds of HIV-1 infection, with regimens including integrase strand transfer inhibitors (INSTIs) achieving viral control (<50 copies/ml) in >65% of PLWH within 4 weeks (12) and increasing to >80% of individuals within 12 weeks (12, 13). Viral RNA decay is at least bi-phasic, with an initial steeper decline reflecting the loss of HIV-1 infected cells that are short-lived (t^1^/_2_–hours to days) and a second slower decline reflecting loss of longer-lived HIV-1 infected cells (t^1^/_2_–weeks to years) (14–18). In most individuals, peripheral CD4^+^ T cell counts increase significantly within 4 weeks of ART initiation and are restored to levels comparable with uninfected individuals in ~12 months (13). ART also significantly reduces virus-driven immune activation (19), though levels of cellular activation and some proinflammatory cytokines remain higher than in HIV-1 seronegative individuals [reviewed in (20)]. Individuals with durably suppressed viral load on ART in North America and Europe now enjoy near-normal lifespans (21, 22). Yet, with the notable exception of two case reports of long-term remission (23–25), HIV-1 infection cannot be cured using current regimens.

In durably suppressed (>12 months) individuals, the barrier to HIV-1 cure is a rare population of HIV-1 infected cells that are not producing virions (are therefore impervious to the host immune system) but can reactivate to produce replication competent virus. Collectively, these cells are referred to as the latent HIV-1 reservoir (26) and, following interruption of ART, are the source of virus rebound. HIV-1 rebound is consistently observed after 1–12 weeks of ART withdrawal, irrespective of whether ART was initiated in the first weeks–months of infection or years later in chronic infection (27–29). Rebounding virus is typically oligoclonal, suggesting reactivation of >1 latently infected cell (28, 30).

The best-characterized portion of the latent reservoir resides in CD4^+^ T cells (31, 32). In ART-treated people, >95% of HIV-1 proviruses encode a viral genome that is replication incompetent (4, 5). However, replication competent proviruses can be reactivated following mitogenic stimulation of cells *in vitro* and quantified by measurement of viral RNA or viral antigen. Finzi et al. showed that in most individuals resting CD4^+^ T cells (not expressing markers of cellular activation) produce replication competent HIV-1 following mitogenic stimulation (33). The frequency of cells harboring inducible, replication competent virus is very low, ~1 infected cell/10^6^ resting CD4^+^ T cells (33) [range: 0.01–10 infected cells/million resting CD4^+^ T cells (34)] but is remarkably stable in ART-suppressed individuals. Two independent longitudinal studies showed that the half-life of the measured reservoir is 44 months (34, 35). These data can best be explained by ART-suppressed individuals harboring a small population of HIV-1 latently infected CD4^+^ T cells that are long-lived and/or undergo homeostatic proliferation and undergo occasional stochastic reactivation. Note, while replication competent virus can also be recovered from resting CD4^+^ T cells in untreated infection, the frequency of infection is >2 logs higher and correlates directly with plasma virus load (15), suggesting that these resting CD4^+^ T cells harbor contemporaneous viruses (similar to those in the plasma) and do not represent a stable reservoir.

To date, efforts to cure HIV-1 in ART-treated participants have been unsuccessful. Early ART treatment both in non-human primates 3 days after infection with a simian immunodeficiency virus (SIV) (36) and HIV-1-infected people (37–39) has not prevented reservoir formation. In addition, latency reversing agents that seek to force HIV-1 reactivation have induced transient blips of detectable virus in the plasma, but have not impacted the size of the stable replication competent reservoir (40). The intractable nature of the HIV-1 reservoir has led to support for “functional cure” strategies that do not entirely eradicate HIV-1 but rather constrain viral rebound following ART interruption (28). Given that strategies to purge or suppress the stable HIV reservoir have had limited success, it is worth considering whether it is possible to limit the size of the reservoir by blocking its formation.

## The IL-7/IL-7Rα (CD127) Pathway Is a Critical Regulator of CD4^+^ T Cell Homeostasis

In both humans and animal models, the primary T cell response to infection is dominated by, antigen-specific T cells that are strongly proliferative (Ki67^+^), express activation markers including CD69 and CD25 (in humans, HLA-DR), but mostly are short-lived. As the acute antigen load decreases, the bulk of primary activated T cells undergo apoptotic death and the effector T cell pool contracts (41, 42). A subset of cells survive (42, 43), having undergone changes that enable them to become long-lived and persist in the absence of antigen. These memory cells retain proliferative potential including homeostatic proliferation (41, 42, 44–46). Memory T cells are responsible for mediating ongoing immune surveillance. In humans, both vaccine studies and BrdU (synthetic nucleoside) labeling of proliferating T cells [reviewed in (47)] have identified subpopulations of memory T cells with half-lives as long as 9 years (48, 49). In response to secondary antigen stimulation, memory T cells exhibit rapid proliferation and give rise to both short-lived effector memory and terminally differentiated effector T cells. In humans who are exposed to many different pathogens, including chronic infections like HIV-1 in which antigen stimulation is ongoing, both short-lived effector and long-lived memory T cells co-circulate.

Short- and long-lived antigen-specific T cells can be further delineated based on their homing capability, anatomical location, phenotype and function which collectively reflect lineage differentiation. Stem cell-like (TSCM) and central (TCM) cell phenotypes harbor a high proportion of long-lived cells whereas transitional (TTM), effector (TEM) and terminal effector (TEMRA) cell populations are more short-lived and express higher levels of activation markers (50).

Maintenance of the equilibrium between naïve, effector and long-lived memory T cells (and their lineage subsets) is termed T cell homeostasis. IL-7 is a common γ-chain cytokine that together with IL-2 and IL-15 regulate homeostasis of both CD4^+^ and CD8^+^ T cells, as well as other lymphocytes. IL-7 is constitutively expressed by stromal and epithelial cells in the thymus, lymphoid tissue and bone marrow, and regulates multiple stages of the T cell life cycle including thymopoeisis, memory cell maturation (44), survival (51), and homeostatic proliferation (52–55). IL-7 is not, however, required for the initial primary expansion of activated effector T cells (53).

IL-7 signals through the IL-7R heterodimer which consists of the common gamma chain (CD132), shared by IL-2 and IL-15 receptors, and IL-7Rα (CD127), which confers specificity to IL-7. IL-7/IL-7R engagement induces JAK/STAT signaling which regulates expression of proliferative and anti-apoptopic genes, including increased expression of the Bcl2 anti-apoptotic gene family, promoting cell survival (54, 56). IL-7 binding also reduces CD127 expression on T cells through both transcriptional and post-transcriptional mechanisms. CD127 is expressed at high levels on naïve T cells (52) and TSCM (57) but is downregulated on activated effector T cells (50, 53, 58–60). As cellular activation decreases and T cells transition to long-lived memory, CD127 is re-expressed (44, 50, 53, 61) (Figure 1). In healthy humans with no overt infection, 60–90% of circulating memory (CD45RO^+^) CD4 T cells in the blood [unpublished observations (62, 63)] and 60–80% of resident memory CD4^+^ T cells (CD69^+^CD4^+^CD45RO^+^) in lymphoid, lung, and gut tissues are CD127hi (64). Consistent with CD127 expression patterns and cellular half-lives, Bcl2 expression is high in naïve and memory CD4^+^ T cells but lower in effector T cells (52, 65, 66).

The dynamic nature of CD127 expression on CD4^+^ (and CD8^+^) T cells reflects that IL-7 levels are not regulated by production but by consumption (67). Downmodulation of CD127 in response to IL-7 binding allows available IL-7 to be shared by the greatest number of cells (68, 69). When T cell homeostasis is dysregulated and lymphopenia occurs (e.g., following myeloablative chemotherapy or CD4^+^ T cell depletion following HIV infection), IL-7 consumption declines and serum IL-7 levels increase (70). This excess drives rapid expansion of both CD127^+^ naïve (52) and memory T cells (70) promoting restoration of lymphocyte levels.

## CD127^+^ Memory CD4^+^ T Cells Harbor Replication Competent HIV-1

HIV-1 infects T cells via CD4^+^ and a co-receptor (CCR5 or CXCR4). In untreated infection, HIV-1 infection occurs mostly in effector memory and not naïve CD4^+^ T cells, in part because memory, particularly activated memory (CD127lo), CD4^+^ T cells express higher co-receptor levels (63, 71). By contrast, naïve CD4^+^ T cells typically lack CCR5 (72). Characterization of CD4^+^ T cells harboring latent HIV in ART-suppressed individuals is very challenging due to the low frequency of circulating infected long-lived cells. Primary cell models of HIV latency, in which a much higher frequency of CD4^+^ T are infected have proved informative. In superinfected aggregate cultures of tonsils, CD127^+^CD4^+^ T cells were infected with HIV but did not support viral gene expression (73), suggesting these, CD127^+^ CD4^+^ T cells may promote HIV latency. In another primary cell model of HIV-1 latency, in which CD4^+^ T cells were derived from PBMC, CD127 expression was highly associated with latent infection (74). Shan and colleagues also employed primary cell models to show latent HIV infection (as opposed to productive infection), preferentially occurs at the transition of CD4^+^ T cells from an effector to a memory state (75). Transcriptional reprogramming of CD4^+^ T cells from the effector to memory state, which was marked by high CCR5 expression, facilitated HIV-1 integration but not subsequent HIV-1 gene expression, thereby promoting latency (75). In summary, HIV-1 CD127^+^ CD4^+^ memory T cells may be more likely to harbor persistent HIV-1 with establishment occurring at the transition of activated effector (CD127lo) to longer-lived memory (CD127hi) CD4^+^ T cells.

## HIV-1 Infection Dysregulates CD4^+^ T Cell Homeostasis

HIV-1 infection creates multiple challenges for CD4^+^ T cell homeostasis, most obviously reflected in the absolute loss of CD4^+^ T cells in untreated infection The cytopathic effects of direct CD4^+^ T cell infection alone do not explain this loss of CD4^+^ T cells, suggesting indirect mechanisms (76). A major contributor to CD4^+^ T cell depletion in acute and chronic infection is generalized immune activation driven by unabated HIV-1 viremia that can reach 10^8^ copies/ml during acute infection and typically remains >10^4^ copies/ml in chronic infection (77). These unusually high and sustained antigen levels in turn, induce sustained elevation of activation and exhaustion markers on CD4^+^ T cells (78, 79). This is associated with diminished IL-2 release by CD4^+^ T cells (58, 80), increased peripheral turnover of both naïve and memory CD4^+^ T cells and critically, failure to generate long-lived memory CD4^+^ T cells (81–84).

Dysregulated IL-7/IL-7R signaling in HIV-1 infection (85) has been proposed by several groups as a critical link between HIV-1-driven immune activation and bystander CD4^+^ T cell loss (86–88). Firstly, activation-induced lymphodepletion increases serum levels of IL-7 (89), combines with other proinflammatory cytokines, such as IL-1β [which is elevated in the lymphoid tissues of HIV infected individuals (88)], to increase turnover of antigen-specific CD4^+^ T cells favoring the generation of short-lived CD127lo/-activated effector T cells (84, 90, 91). Although circulating IL-7 levels rise, IL-7 bioavailability in the lymphoid tissues is significantly decreased following infection due to TGF-β1-mediated collagen deposition that results in the loss of IL-7-producing fibroblast reticular cell (FRC) networks (92, 93). This is proposed to directly contribute to the increased apoptosis and loss of naïve CD4^+^ T cells that mostly reside in lymphoid tissue (93, 94).

The overall effect of these changes is impairment of both the generation and maintenance of long-lived CD4^+^ T cells in viremic individuals. Several groups have reported both significantly lower frequencies of CD127^+^CD4^+^ T cells as well as lower CD127 expression levels on CD4^+^ T cells in untreated HIV-1 infection (50, 62, 86, 89, 95, 96). Notably, expression of the CD132 common γ chain remains normal on CD4^+^ T cells in infected individuals, suggesting a specific impact on IL-7 signaling on CD4^+^ T cells (97). Down-regulation of CD127 on T cells correlated significantly with both depletion of absolute levels of CD4^+^ T cells and also with increased concentration of serum IL-7. The decreased CD127 expression was associated with lower cellular levels of Bcl-2 and with the poorer survival of T cells in the presence of IL-7 *in vitro* (86). CD127^+^CD4^+^ T cells also exhibited increased rates of apoptosis in untreated infection relative to healthy controls suggesting in untreated infection, CD127 expression on memory cells is not itself sufficient to maintain cell survival in the face of uncontrolled HIV-1 viremia (89, 98). By contrast, HIV-1 infected individuals who exhibited long term non-progression had higher CD127^+^CD4^+^ T cell frequencies than HIV-1 infected typical progressors (62, 99) and in some individuals, decreased CD127 expression on CD4^+^ T cells preceded subsequent loss of virus control (62).

The loss of CD127^+^CD4^+^ memory T cells in untreated HIV infection is reflected in lower antigen-specific CD4^+^ T cell responses to chronic infections. Frequencies of Cytomegalovirus (CMV) (100) and *Mycobacterium tuberculosis* (*M.tb*)-specific CD4^+^ T cell responses (101) are significantly lower in HIV infected individuals relative to healthy individuals and, despite clearly detectable HIV-specific CD8^+^ T cell responses, little to no HIV-specific CD4^+^ T cell proliferation is detectable in untreated HIV infection (100). Generally, immune responses to vaccination (humoral and cellular) are lower and less durable in HIV infected individuals compared to healthy individuals, suggesting weaker CD4^+^ T cell help [reviewed in (102, 103)]. In one study, vaccination with the experimental vaccine Modified Vaccinia Ankara (MVA) expressing the *M.tb* antigen, 85A was compared in HIV uninfected, HIV viremic (CD4^+^ count >300 cells/mm^3^) and ART-suppressed individuals. Consistent with the formation of long-lived T cell memory being limited during untreated HIV-1 infection, vaccine-induced oligofunctional CD4^+^ T cell responses at peak and over the course of the following year were significantly lower in untreated HIV-1 infected participants relative in uninfected and HIV infected, ART-treated study participants (101).

In summary, uncontrolled HIV-1 infection skews the memory CD4^+^ T cell response to a short-lived effector phenotype with lower frequencies of long-lived memory CD4^+^ T cells, suggesting either or both impaired effector to memory transition of CD4^+^ T cells or a failure to maintain long-lived memory CD4^+^ T cells. Dysregulated IL-7/IL-R signaling appears central to these changes.

## ART Restores the CD4^+^ T Cell Memory Transition

As described above, as a pathogen is cleared the population of activated, short-lived effector T cells contracts and quiescent, longer-lived pathogen-specific memory cells emerge. A similar phenomenon but on a broader scale, impacting both HIV-specific and non-specific CD4 T cells, is observed in the weeks to months following ART initiation.

Successful ART (91) rapidly reduces viremia. Immune activation is significantly reduced, but not fully abrogated, possibly because of residual low-level viremia. Elevated turnover of CD4^+^ T cells is decreased to levels that are comparable with healthy controls (81, 104) within 12 weeks of ART (39, 77). Both absolute CD4^+^ T cell counts and CD127^+^ CD4^+^ memory T cell frequencies increase to levels observed in uninfected individuals (105), though CD127 expression levels on CD4^+^ T cells remain lower (106). With restoration of absolute CD4^+^ T cell levels, IL-7 in the serum decreases and IL-7 mediated STAT-5 phosphorylation, which is elevated in memory CD4^+^ T cells in untreated infection (107), is normalized (108). Functionally, memory CD4^+^ T cells exhibit improved IL-2 release, HIV-1-specific CD4^+^ T cell responses increase in frequency (100, 109) and CD4^+^ T cell responses to vaccination improve (101). By comparison, immunological non-responders to ART, in whom viremia is controlled but absolute CD4^+^ T cells counts are not fully restored, have higher serum IL-7 levels and lower CD127^+^CD4^+^ T cells compared with immunological responders (105, 106, 110). Altogether, in most people ART largely restores CD4^+^ T cell homeostasis, including CD4^+^ T cell transition from effector to long-lived memory T cells.

## Therapeutic Implications of ART-Mediated Restoration of CD4^+^ T Cell Homeostasis

**Figure 2:**
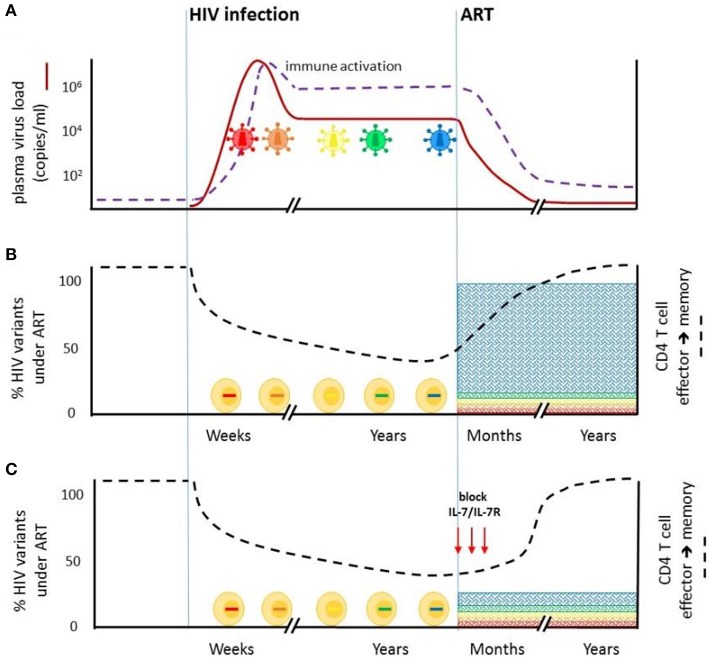
Blockade of IL7/IL-7R signaling at the time of ART initiation may limit the size of the stable HIV-1 reservoir. **(A)** HIV-1 infection is characterized by extensive viral replication (red line) and virus evolution (colored virions). Virus-driven immune activation (purple dotted line) is observed throughout untreated infection. ART rapidly reduces plasma virus levels. Immune activation is also significantly reduced but not fully abrogated. **(B)** HIV-1 infection impairs CD4^+^ T cell effector to memory transition (black dotted line), which is restored on ART. Abrahams et al. (2) showed that on average, 80% of the replication-competent virus in CD4^+^ T cells from durably ART-suppressed individuals is derived from virus present in plasma in the year prior to ART initiation. Viruses that were circulating earlier in untreated infection comprise a minority of the latent reservoir. **(C)** Blocking IL-7/IL-7R signaling at the time of ART initiation, aimed at delaying ART-mediated restoration of CD4^+^ T cell effector to memory transition, may limit the entry of viruses circulating immediately prior to ART into the HIV reservoir.

While current curative strategies (eradication or functional) against HIV-1 largely target the established stable HIV-1 reservoir in durably suppressed individuals, we propose that strategies to limit the seeding of long-lived latently infected cells at the time of ART will likely decrease the size of the reservoir. We propose targeting the IL-7/IL-7R pathway by specifically blocking CD127 signaling on CD4^+^ T cells in early ART to delay restoration of the CD4^+^ T cell memory transition (Figure 2).

Monoclonal antibodies (MAb) that antagonize the IL-7Rα (111) are in investigation for treatment of a range of autoimmune diseases and inflammatory conditions, including diabetes (112), multiple sclerosis, rheumatoid arthritis (113, 114), and inflammatory bowel disease (115). The aim of these approaches is to suppress aberrant memory CD4^+^ T cell responses (116). A single intravenous administration of anti-CD127 antagonist antibody resulted in inhibition of antigen-specific memory CD4^+^ T cell responses and decreased chronic inflammation in primates that was sustained for 11 weeks (111). In diabetes studies in mice, CD127 blockage decreased T helper 1 (TH1) IFN-γ-producing CD4^+^ T cells in secondary lymphoid tissues (117) and elevated PD-1 expression on autoreactive CD4^+^ T cells, limiting long-memory responses (118). In other murine studies, anti-CD127 antibodies blocked memory CD4^+^ T cell proliferation and blunted vaccine-induced immune responses (119).

In humans, a single intravenous administration of an αCD127 antagonist mAb was well-tolerated (120) with both full receptor occupancy and inhibition of IL-7/IL-7 signaling (*ex vivo* STAT5 phosphorylation) observed for over 21 days following dosing. Transient depletion of CD19^+^ B cells and limited changes to T cells, including T regulatory cells (discussed below), were observed during the 24 weeks of participant follow-up. Expression of the activation/exhaustion marker, PD-1 on T cells did not consistently change following dosing. In summary, anti-CD127 immunotherapy is a promising approach to limit CD4^+^ T cell memory immunity that in clinical studies to date is supported by pharmokinetic and safety data (120).

While we propose blocking CD127 in combination with ART initiation to limit reservoir establishment, others had proposed IL-7 treatment in PLWH on ART as a strategy to improve CD4^+^ and CD8^+^ T cell immunity to HIV-1, particularly in individuals who do not regain normal CD4^+^ T cell counts after virologically successful ART. Consistent with other IL-7 immunotherapy studies (121, 122), treatment of durably ART-suppressed individuals successfully increased circulating CD4^+^ and CD8^+^ T cell counts for several months after last dosing (123–126) by increasing expression of pro-survival genes (127). However, the effect on the HIV reservoir was also to increase the frequency of CD4^+^ T cells containing HIV-1 (124, 128), possibly by CD95-mediated proliferation (129). These results complement our hypothesis that that CD4^+^ T cell homeostasis mediated by IL-7/IL-7R signaling is critical for the establishment and maintenance of the long-lived latent HIV-1 reservoir.

INSTIs are now widely included as a first-line therapy against HIV-1 largely because of good tolerability (130). INSTI containing regimens produce ~1 log greater decrease in VL within the first 10–15 days following ART initiation (18). This more rapid decrease in antigen levels produces proportionate and earlier decreases in cellular immune activation (131) that are likely to result in earlier T cell memory restoration, arguably within days. Accordingly, we propose that blocking of T cell memory formation to prevent HIV-1 seeding of the reservoir should begin very early, possibly alongside ART initiation and continue short-term until all productively infected CD4^+^ T cells are cleared; that is the participant is no longer viremic (Figure 2). In the pre-INSTI era, ART regimens increased CD127^+^CD4^+^ CM T cells within 1 month of ART initiation (132). Detailed studies describing the kinetics of CD4^+^ T cell memory restoration in the weeks-months following INSTI-ART initiation are however needed to better inform dosing strategies.

IL-7 is also critical for memory T cell homeostatic proliferation. While this perspective prioritizes employing IL-7R blocking to delay CD4 T cell restoration at ART initiation, CD127 antagonism may have application in limiting homeostatic proliferation of latently infected cells under durable ART (8). Here, bi-specific antibody approaches to express cis-acting antibodies [reviewed in (133)] targeting both CD127 and membrane-bound HIV proteins may increase specificity and facilitate longer-term treatment.

## Specific Considerations/Limitations

There are number of considerations and limitations to this perspective to HIV cure.

1. Firstly, antagonizing CD127 signaling at ART initiation will not block HIV-1 already integrated into long-lived CD4^+^ T cells. Both studies by Brodin et al. (3) and Abrahams et al. (2) as well as another smaller study by Jones et al. (134) found that HIV-1 variants from much earlier in infection were genetically similar to a subset of post-ART viruses. Further work is required to understand the mechanism/s by which HIV-infected cells harboring these viruses were able to persist during long periods of untreated infection. One possibility is that while HIV-1 infection impairs CD4^+^ T cell memory formation, this is incompletely abrogated and a small number of cells infected early in untreated infection may become long-lived memory CD4^+^ T cells despite profound dysregulation in IL-7 signaling at the population level (Figure 1).
2. The observations of Brodin et al. (1) and Abrahams, Joseph et al. (2) were made in peripheral CD4^+^ T cells. HIV reservoirs are not limited to the blood. Recent studies have identified T follicular helper cells (TFH) (135) that reside in lymph nodes as the major CD4^+^ T cell subset for HIV infection and replication in PLWH (136, 137) that continue to serve as a persistent HIV reservoir in PLWH on ART (138, 139). Whether replication competent viruses in TFH also cluster with viruses circulating around the time of ART, suggesting TFH could also be targeted by CD127 blocking, requires investigation. Similar studies are needed to examine other tissue reservoirs. Here, animal models of HIV infection and persistence will be particularly useful.
3. While α-CD127 antibody therapy has been well-tolerated in healthy individuals [(120) NCT02293161, NCT02293161], treatment of PLWH must be evaluated for risks associated with delayed recovery of CD4^+^ T cell homeostasis [immunological non-responders (140)], particularly in individuals with advanced disease and/or low CD4^+^ nadir (141).
  First-line INSTI ART regimens are associated with a higher incidence of immune reconstitution immunodeficiency syndrome (IRIS) (142, 143). IRIS, that can worsen existing opportunistic infections or unmask previously subclinical infections, very commonly *M.tb*, is associated with redistribution and restoration of functional memory T cells within the first months of ART (144). A single dose of α-CD127 antagonist mAb in NHP produced significant and prolonged decreases of IFN-γ secreting *M.tb*-specific CD4 T cells without CD4^+^ T cell depletion (111). It is attractive to speculate whether short-term α-CD127 antibody therapy when given in combination with ART-initiation to PLWH, could also afford some protection against IRIS-associated events.
4. T regulatory (Treg) CD4^+^ T cells function to suppress potentially deleterious activities of other T helper cells particularly TH1 and TH17 cells. Like other CD4 T helper cell subsets, Treg CD4^+^ T cells express CCR5, are readily infected in untreated HIV infection (145) and in PLWH on ART, can harbor replication competent virus (139); with some reports that Treg CD4^+^ T cells are enriched for latent HIV relative to conventional T helper subsets [reviewed in (146)]. Treg CD4^+^ T cells differ from other conventional CD4^+^ T helper subsets in that they are CD127lo (147). IL-7 can however induce STAT-5 phosphorylation in these cells in a dose-dependent manner (115). Following ART initiation, Treg CD4^+^ T cells frequencies increase further in the first week of ART initiation then decrease to normal ranges in most individuals (148). The effect of CD127 antagonism on Treg CD4^+^ T cells appears different in NHP and mice models. In NHP, CD127 blocking did not increase PD-1 expression (111) but increases in PD-1 expression as well and increases Treg CD4^+^ T cells frequencies were observed in mice (112). Further studies are needed, particularly to investigate if outgrowth viruses from Treg CD4^+^ T cells cluster with early or late (pre-ART) viruses. Treg CD4^+^ T cells in PLWH however represent a cell subset that may be refractory to IL-7/IL-7R blocking strategies following ART initiation (112).
5. A CD127 blocking strategy will impact the signaling of all CD127 expressing cells. This includes naïve CD4^+^ T cells, naïve and memory CD8^+^ T cells (111), γδ T cells and innate lymphoid cells (ILCs) including NK cells. In humans, clinical administration of an anti-CD127 antagonist antibody induced minimal changes beyond short-term B cell loss (120), consistent with observation in NHP in which anti-CD127 mAbs produced no changes in peripheral T and B cell frequencies, nor changes in T cell subsets including Treg cells (111). In that study, CD127 antagonism did not increase PD-1 levels on CD8^+^ T cells (111), however, studies in humanized mice using the same antibody clone increased the exhaustion signature particularly Tim-3 and PD-1 on CD8^+^ T cells (115). Given the disparities in animal studies, detailed functional studies are needed in humans to examine the impact of CD127 antagonism on lymphocyte subsets, particularly cytolytic, subsets. Non-cytolytic ILCs do not recover on ART (149) and similarly, studies are needed to investigate whether there are any additional, deleterious effects of CD127 antagonism on this cell subset (150).
6. While resting CD4^+^ T cells constitute the largest HIV-1 reservoir in the body, other CD4^+^ expressing cells, such as macrophages, can harbor HIV-1 and may contribute to virus rebound (151). Much less is understood about formation of HIV latency in these cell subsets. Macrophages express CD127 and antibody blocking of this pathway increases autophagy (152). How this process would impact the HIV persistence is unclear. γδ T cells have been shown to harbor replication competent HIV in PLWH on ART (153) and also express CD127. Future studies will need to investigate how CD127 blocking modulates HIV persistence in non-CD4^+^ T cell subsets.

## Summary

We propose that restoration of CD4^+^ memory transition in ART treated participants, which enables the generation of long-lived CD4^+^ T cells, drives the majority of HIV-1 reservoir formation. A temporary blockade of IL-7/IL-7R signaling at the time of ART initiation, by delaying memory CD4^+^ T cell restoration until virus has been cleared, could limit the size of the stable HIV reservoir, facilitating HIV-1 cure efforts. Limiting the size of the long-lived reservoir could also be combined with other strategies aimed at minimizing homeostatic proliferation of memory CD4^+^ T cells harboring HIV by limiting CD4^+^ T cell proliferation (154) or strategies to further reduce the established HIV reservoir following latency reversal and immune-mediated clearance. It is likely a combination of HIV cure strategies will be required to enable long-term ART interruption without virus rebound.

## Data Availability

The datasets generated for this study are available on request to the corresponding author.

## Author Contributions

NG conceived the manuscript. All authors contributed to the writing of the manuscript.

### Conflict of Interest Statement

The authors declare that the research was conducted in the absence of any commercial or financial relationships that could be construed as a potential conflict of interest.
